# Specialized Metabolic Reprogramming in Plant Immunity: Biosynthetic Networks, Spatiotemporal Regulation, and Quantitative Defense

**DOI:** 10.3390/plants15101424

**Published:** 2026-05-07

**Authors:** Adnan Amin

**Affiliations:** Department of Life Sciences, Yeungnam University, Gyeongsan 38541, Republic of Korea; adnan.amin@yu.ac.kr

**Keywords:** spatiotemporal compartmentation, metabolomics, plant immunity, specialized metabolism, growth–defense trade-off

## Abstract

Specialized metabolic reprogramming is a central component of plant immunity. However, its integration across biosynthetic networks and defense phenotypes remains incompletely understood. This mini review examines how specialized metabolites are produced, regulated, spatially deployed, and linked to defense outcomes. We highlight how metabolites such as camalexin, indolic glucosinolates, benzoxazinoids, flavonoids, lignin precursors, pipecolic acid, and N-hydroxypipecolic acid are produced through pathway branching, metabolic flux redistribution, and coordination with primary metabolism. We further discuss how immune signaling modules and transcriptional regulators, including salicylic acid, jasmonic acid, and ethylene pathways, together with transcription factors, regulate defense mechanisms through genes such as PAD3, CYP71A12, CYP71A13, ALD1, SARD4, FMO1, JAZ, and ORA59. Emphasis is placed on spatiotemporal compartmentation, including cell- and tissue-specific responses, plastidial and endoplasmic reticulum-associated metabolism, vacuolar sequestration, apoplastic deployment, and transport-dependent localization, as metabolite function depends greatly on when and where compounds accumulate. This review also evaluates how these metabolic programs are translated into quantitative defense phenotypes, such as resistance outcomes, growth–defense tradeoffs, and fitness costs. Finally, we evaluate emerging tools, including metabolomics, spatial metabolomics, multiomics integration, network inference, and predictive modeling, to elucidate causal relationships between metabolic reprogramming and immune performance. Collectively, the evidence supports a multiscale framework in which specialized metabolism links immune perception to quantitative defense output.

## 1. Introduction

Plant immunity requires rapid metabolic reprogramming following a pattern and effector-triggered recognition. In addition to canonical signaling events, including calcium influx, mitogen-activated protein kinase cascades, and defense hormone accumulation, resistance depends on specialized metabolites that function in processes such as antimicrobial defense, redox regulation, structural reinforcement, and ecological interactions [1]. However, plant resistance is also strongly shaped by the regulatory and defensive functions of specialized metabolites [2,3]. These observations support the view that specialized metabolism is not a peripheral output of immunity but rather a central component of immune execution and adaptation [4].

It has been well established that plant defense metabolism is not organized as a single, isolated linear pathway that produces defined outcomes, but rather comprises complex, interconnected biosynthetic pathways. This complex interconnection of pathways is normally framed with combinatorial regulation across hierarchical control layers, precursor competition, pathway branching, and flux redistribution [5]. Among the precursors, the TCA (tricarboxylic acid cycle), glycolysis, carbon metabolism, and salicylic acid (SA) pathways are important in linking resource allocation directly to defense mechanisms [6]. Consistent with this view, a typical example includes integrated transcriptomic and metabolomic analyses of maize defense mutants. The coordinated activation of phenylpropanoid, flavonoid, lignin, and terpenoid biosynthesis, along with repression of photosynthetic functions, was observed. This clearly illustrates that specialized defense output emerges from system-wide metabolic reallocation rather than via single-pathway induction [7].

A multilayered complex regulatory system is primarily involved in regulating biochemical plasticity in plant defense, involving an integrated signaling, posttranslational, and transcriptional control [5]. In this complex mechanism, the role of phytohormones, including jasmonic acid (JA), SA, and ethylene (ET), is essential, as they regulate “pathway prioritization”. Moreover, diverse transcription factors, including MYB and bHLH, modulate metabolic gene expression and the development of specialized cells, which support defensive chemistry [8]. Protein kinases also participate directly in specialized metabolic reprogramming by transducing the stress signals into downstream biosynthetic activation and posttranslational regulation [9]. In parallel, glycosylation and deglycosylation reactions modify the stability, mobility, storage, and biological efficacy of immune-related small molecules, thereby reinforcing that metabolite function is dependent on the relative abundance and biochemical state [10]. These observations support a view of plant defense metabolism as a dynamic regulatory system in which pathway architecture, signaling logic, and metabolite modification are tightly coupled.

In plant immune responses, another important layer is spatiotemporal organization, which is crucial for immune functioning. Several adaptive mechanisms largely promote the activity of plant metabolites. Based on the time and site of accumulation, these specialized metabolites act on the subcellular, cellular, tissue, and organ scales to produce defined ecological functions. Recently, it has been observed (e.g., in root immunity) that defense processes must be transcribed at relevant spatial and temporal scales. This is because tissue microenvironments, microbial exposure, and host responsiveness vary considerably diagonally in the plant body [11]. The advanced spatial workflows and mass spectrometry-based metabolomic approaches provide a powerful foundation to capture these distributions [12,13]. However, these workflows vary in annotation depth, platform coverage, and interpretive strength. Therefore, linking metabolite accumulation to quantitative defense phenotypes requires more than descriptive profiling. It presents a causal resolution of the key questions, including the site of biosynthesis [3], the transport of metabolites, and, importantly, the localized pool through which they contribute toward resistance.

There is a clear research gap due to the lack of a coordinated framework that links biosynthetic network architecture, regulatory control, spatiotemporal compartmentation, and quantitative defense phenotypes. In the literature, several studies have focused on either regulatory modules or plant-defense-related metabolites. Similarly, others have highlighted the regulation of resistance, growth costs, and fitness tradeoffs through network topology, tissue specificity, and metabolic states. In this review, specialized metabolic reprogramming is considered across the major categories of plant immune challenges, including biotrophic, hemibiotrophic, and necrotrophic pathogens, herbivores, and root-associated microbial interactions. These challenges differ in their signaling priorities, spatial deployment, and quantitative metabolic outputs and therefore provide an important framework for interpreting the biosynthetic network architecture. In addition, root-associated immunity operates under distinct spatial and ecological constraints, where microbial exposure and tissue-specific responses shape the localized metabolic organization. Therefore, immune metabolism should be interpreted as a context-dependent system rather than a uniform stress response.

## 2. Literature Search Strategy

This review was developed using a targeted literature survey to synthesize current knowledge on specialized metabolic reprogramming in plant immunity. Relevant publications were identified using Web of Science, Scopus, PubMed, and Google Scholar. The search considered studies published in English between 2000 and 2026, with an emphasis on recent advances (the last 6 years), while retaining earlier reports that were essential for the conceptual and mechanistic context. Search combinations included terms such as “plant immunity,” “specialized metabolism,” “defense metabolites,” “phytoalexins,” “flavonoids,” “camalexin,” “pipecolic acid,” “N-hydroxypipecolic acid,” “metabolic reprogramming,” “biosynthetic networks,” “spatial metabolomics,” “multiomics,” and “growth–defense tradeoffs.” Priority was given to primary studies that provided mechanistic, genetic, metabolomic, spatial, or system-level evidence. In contrast, review articles were used selectively to support the conceptual framing and cross-study interpretation. The final literature set was curated based on the publications’ direct relevance to biosynthetic network architecture, regulatory control, spatiotemporal compartmentation, quantitative defense phenotypes, and emerging analytical approaches in plant immune metabolism.

## 3. Biosynthetic Network Architecture of Specialized Metabolites

Defense-associated specialized metabolites originate from interconnected biosynthetic systems rather than from isolated linear pathways [14]. In plant immunity, the organization and deployment of these biosynthetic networks depend on the nature of the immune challenge because biotrophic, hemibiotrophic, and necrotrophic pathogens, herbivores, and root-associated microbial interactions impose distinct regulatory and physiological requirements. These different challenge contexts influence hormone prioritization, precursor allocation, pathway branching, and spatial deployment of the specialized metabolites [15,16]. In particular, hormone crosstalk and signaling hierarchy determine the pathway activation and metabolic flux redistribution under different biotic stresses [17]. Plant immune metabolism should therefore not be interpreted as a uniform stress response but rather as a context-dependent biochemical architecture linked to specific classes of biotic challenges.

### 3.1. Classes of Immune-Related Specialized Metabolites

Depending on the plant species, diverse immune-related metabolite classes have been reported, including phytoalexins, terpenoids, flavonoids, and amino acid-derived immune metabolites. Among these, phytoalexins are a major class of immune-related metabolites that are commonly reported. Camalexin (phytolaxin) is a key characterized example from *Arabidopsis* that is involved in several regulatory pathways (indolic glucosinolate) and is directly related to the synthesis of defense-activated indole derivatives [18,19]. In Brassicales, glucosinolates are designed to contribute toward defense and overall plant growth regulation via their compounds and catabolites [20,21].

**Figure 1:**
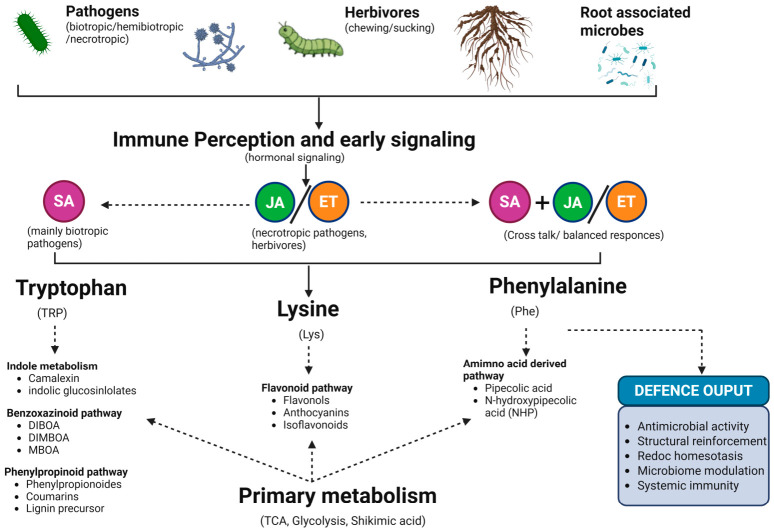
Biosynthetic network architecture and stress-driven flux redistribution in plant immunity. Primary metabolism supplies the precursor pools of tryptophan, phenylalanine, and lysine, which are redirected into the interconnected specialized metabolic pathways. Tryptophan supports the production of camalexin, indolic glucosinolate, and benzoxazinoid biosynthesis; phenylalanine feeds the production of phenylpropanoid-, flavonoid-, and lignin-related branches; and lysine contributes to the production of pipecolic acid and N-hydroxypipecolic acid. Under stressed conditions, flux redistribution across these branches shapes the defense-associated metabolic output.

Benzoxazinoids are commonly found in grasses and primarily facilitate plant defense against pathogens and rhizosphere interactions. Similarly, phenylpropanoids and their derivatives are commonly reported in almost all plant species and provide substantial support for antimicrobial mechanisms and plant cell wall integrity maintenance [22,23]. In parallel, N-hydroxypipecolic acid and pipecolic acid, as lysine derivatives, primarily serve as immune-amplifying metabolites, thereby connecting plant defense machinery with systemic acquired resistance [24,25]. Moreover, flavonoids are considered key metabolites that are produced via the phenylpropanoid pathway and accumulate in a tissue- and stress-specific manner [26]. Various types, including akuranetin, medicarpin, 4′-O-methylated isoflavonoid, vitexin/isovitexin, and luteolin-7-O-glucoside, have been reported as primarily being involved in the accumulation of phytoalexin, redox buffering, antimicrobial activity, and plant–microbe signaling [27,28,29,30]. Collectively, these classes demonstrate that plant defense metabolism primarily comprises “direct toxic compounds” and “signaling active” metabolites that are incorporated within shared biosynthetic machinery (Figure 1).

### 3.2. Complexity of Pathways and Network Connectivity

The plant defense metabolic machinery is largely composed of complex, branched pathways. However, key precursors of the chemically diverse metabolites have shared precursor pools that are distributed among the competing routes. A new immune challenge considerably alters this allocation, and this decision is generally reliant on the environmental conditions, type of pathogens, and developmental stages [5,31]. One characteristic example is tryptophan metabolism, whose flux is redirected toward indols (glucosinolates), camalexin, and other indolic defense metabolites, and undergoes over-classified transcriptional control and condition-dependent enzymatic activities [18,19].

In addition, various key parameters, including glycosylation, substrate channeling, and transitional detoxification, are important in shaping the plant metabolic flux and defense [32]. Various molecular investigations have revealed that operative defense output is primarily dependent on the compatibility of the signaling network, rather than on the presence of a single gene. This phenomenon is observed in benzoxazinoid biosynthesis, where it was noted that the overall performance of pathways can be limited by poor channeling and transfer of intermediates into the endogenous detoxification routes [22]. Furthermore, network connectivity extends beyond the plant cell due to extensive detoxification by pathogens and the microbiota [33,34]. Therefore, defense metabolites should be viewed as nodes within dynamic biochemical and ecological networks.

### 3.3. Primary and Specialized Metabolism Coordination

In plants, the synthesis, transport, and regulation of specialized defense metabolism primarily rely on key metabolic streams. Various complex metabolic pathways, including the TCA cycle, glycolysis, and the SA pathways, provide basic carbon skeletons, reducing power, and aromatic amino acid precursors. These result in the development of metabolites that participate in plant defense, thereby linking immune chemistry with fundamental defense metabolisms [3,35]. This integration is particularly apparent for phenylalanine- and tryptophan-derived defenses, as well as for the catabolism of lysine into pipecolic acid and N-hydroxypipecolic acid [25].

Considerable metabolic restructuring results from this coupling. This is evident from the example of maize defense mutants, where enhanced production of phenylpropanoids, terpenoids, etc., can overlap with the domination of photosynthetic and growth-associated processes [7,36]. In addition, the specialized metabolites can stimulate primary plant physiology and growth–defense tradeoffs, as noted in the case of glucosinolates and other related compounds (metabolites) [2,20]. Therefore, it is important to consider that defense-related specialized metabolism in plants should be understood as a regulated extension of primary metabolism pathways rather than an independent biochemical mechanism. Table 1 summarizes the major classes of specialized metabolites involved in plant immunity, together with representative biosynthetic origins, immune-associated functions, and typical biotic contexts.

## 4. Regulatory Control of Specialized Metabolic Reprogramming

A multilayer mechanism in plants regulates specialized metabolic reprogramming during immunity, thereby connecting pathogen perception, pathway activation, and metabolite diversification. The plant defense response, at the appropriate time and amplitude, is integrated with immune signaling, chromatin-level regulation, hormone crosstalk, transcription factor hierarchies, and posttranscriptional control by these layers. Thus, it is essential to regulate this phenomenon to understand the types of metabolites produced and when, where, and to what extent they shape defense phenotypes.

### 4.1. Immune Signaling Inputs and Pathway Activation

Specialized metabolic pathways are activated downstream of the core immune signaling modules that are triggered by pathogen recognition [37]. Pattern-triggered immunity and effector-triggered immunity initiate calcium influx, reactive oxygen species production, mitogen-activated protein kinase cascades, and defense hormone biosynthesis, thereby reprogramming the metabolic output toward protective chemistry [38]. In defense against hemibiotropic and biotropic pathogens, SA is of utmost importance, whereas plant responses toward herbivores and necrotrophs are primarily directed by JA and ET [39,40], though considerable overlap and context dependence may occur. These signaling mechanisms tend to direct metabolic flux toward defense programs rather than toward metabolic activation.

In the context of phytohormones, the JA pathway represents an immune-metabolic activation model. The JA signaling (perception) at the receptor site releases JAZ-mediated repression of MYC transcription factors, which facilitates several pathway-specific metabolic responses, such as aromatic amino acid-derived defenses and other multiple specific metabolite pathways [41,42]. Similarly, ORA59 (ERF-family regulator) integrates JA and ET signals to regulate JA/ET-responsive defense genes using a differential promoter binding behavior [43] demonstrates the generation of pathway-specific transcriptional outcomes through signal convergence. Therefore, immune signaling inputs function not only as “on–off switches” but also as quantitative and combinatorial regulators of metabolic pathway choices.

### 4.2. Transcriptional and Posttranscriptional Regulation

One of the major factors of specialized metabolic reprogramming is transcriptional control. Various transcription factors, including NAC, WRKY, bHLH, MYB, AP2/ERF, and bZIP, generally facilitate the development of complicated regulatory hierarchies. Upon further activation, they induce the expression of pathway-specific modifiers, biosynthetic genes, and key transport components [44,45]. Such hierarchical regulation (WRKY33 and MYB51) directly regulates the dynamics of camalexin and related indolic pathways in *Arabidopsis*. This is a demonstration of immune metabolism regulation by structured gene networks rather than independent pathways [19]. Correspondingly, WRKY75 positively modulates ORA59-linked outcomes, thereby regulating JA-mediated defense against necrotrophic fungi [46] of transcription factors in connecting hormone signaling with metabolically appropriate immune effectors.

Nevertheless, this transcriptional layer is further outlined by redox-sensitive nuclear signaling, chromatin modifications, mediator functions, and RNA-level regulations. Moreover, the defense gene-induction kinetics and accessibility are primarily prompted by DNA methylation, histone acetylation, and RNA polymerase-linked pathways. Concurrently, modulation of the transcription fate occurs via the modulation of noncoding RNAs and stress dynamics [47]. The transcription factors are further altered by several posttranslational modifications, including SUMOylation, phosphorylation, and ubiquitination, that can modify the promoters’ stability, activity, and specificity [48,49]. Therefore, it is clear that specialized metabolism is regulated through a multilayer signaling process, in addition to the abundance of transcription factors.

### 4.3. Hormone Crosstalk in Growth–Defense Balance

During plant defense against stress, the decision to include specific metabolic pathways and their expression intensity (as a function of stress) primarily depends on hormonal crosstalk. Various phytohormones, including JA, SA, AA, auxins, gibberellins, and ET, interact in highly interconnected nodes, thereby allowing plants to modify their immune output [16,50]. Among these pathways, a recurring theme is the antagonism between SA and JA/ET signaling, although, in practical terms, this crosstalk is conditional and often fractional [15,51]. Furthermore, abscisic acid (ABA) should be considered in this regulatory framework because it modulates immune signaling and metabolic prioritization in a context-dependent manner [52]. ABA is often associated with stress-adaptive responses, such as stomatal regulation and resource reallocation, but it also intersects with the SA-, JA-, and ET-dependent pathways to influence defense outputs and the associated metabolic adjustments [53]. Thus, ABA contributes to growth–defense balance not as a simple positive or negative regulator but rather as a conditional coordinator of stress and immune metabolism [54]. This relies on the diverse lifestyles, tissues, and developmental stages of pathogens, which can markedly shift the balance of pathway engagement. As phytohormones regulate the production of several specialized metabolites, this crosstalk directly influences defense chemistry.

**Figure 2:**
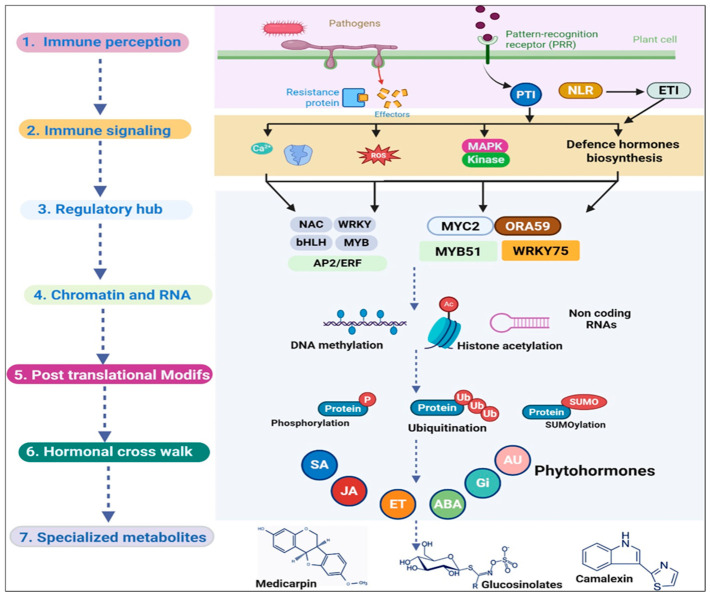
Detailed overview of the regulatory control of specialized metabolic reprogramming during plant immunity involving pathogen perception, core immune signaling module activation, and defense hormone biosynthesis.

This viewpoint is supported by examples from model and crop systems. For instance, in Figure 2, the low-intensity red/far-red conditions lower camalexin and indolic glucosinolate biosynthesis via JA-linked signaling pathways [55]. Therefore, hormonal crosstalk alters quantitative resistance via the regulation of marker genes involved in biosynthesis of specialized metabolites [56] (Figure 2).

## 5. Spatiotemporal Compartmentation of Defense Metabolism

Beyond chemical identity, the defensive function of specialized metabolism depends on where and when the metabolites are synthesized, stored, transported, and activated.

### 5.1. Cellular and Tissue-Specific Metabolic Responses

In plants, defense metabolism and signal perception are heterogeneous across cells and tissues. Different plant parts, such as the mesophyll layer, epidermis, vasculature, and roots accumulate specialized metabolites differently (e.g., their concentrations) and thus induce different immune responses to the perception of pathogens and signal propagation [57,58]. Particularly in roots, as microbial exposure differs across the developmental zones and surface microenvironments, the defense responses must be interpreted at the cellular resolution and over time [11,59]. This spatial heterogeneity likely amplifies differences in local resistance, microbiome assembly, and systemic signaling.

The biological function of defense metabolites depends on their tissue-specific localization and accumulation, including the intracellular, apoplastic, and rhizosphere-associated pools, and cannot be inferred from their abundance alone [60,61,62].

### 5.2. Subcellular Compartmentation and Metabolic Channeling

Different cellular components, including plastids, mitochondria, endoplasmic reticulum, peroxisomes, vacuoles, and the apoplast, provide discrete biochemical environments, which affect precursor access, redox state, and enzymatic localization [63,64]. Such an association is particularly pertinent in plant immunity, as reactive oxygen species, glutathione, and other redox-active metabolites are themselves compartment- and tissue-specific, and their localization determines the downstream defense response [65].

In addition, compartmentation supports metabolic channeling [66]. Although direct evidence remains limited for several defense pathways, the colocalization of enzymes and substrates within defined membrane systems may reduce toxicity and improve the pathway efficiency [67,68]. More broadly, investigations into compartmentalized sugar metabolism and signaling in plants have revealed altered physiological outcomes. This is primarily attributed to the involvement of spatial separation as a major regulatory mechanism rather than a passive structural feature [69].

### 5.3. Sequestration, Transport, and Extracellular Placement of Metabolites

In plants, nature has provided conjugated compartments for the long-term storage of specialized metabolites, protecting them from autotoxicity and enabling fast release thereof just after tissue injury or infection [66,70]. These mechanisms, including membrane trafficking, vacuolar storage, and apoplastic release, determine whether a specialized metabolite functions as a latent reserve, an extracellular defense compound, or an intracellular signaling molecule [71,72]. This is particularly relevant to chemically reactive or unstable compounds whose efficacy depends on controlled release rather than on basic exposure.

**Figure 3:**
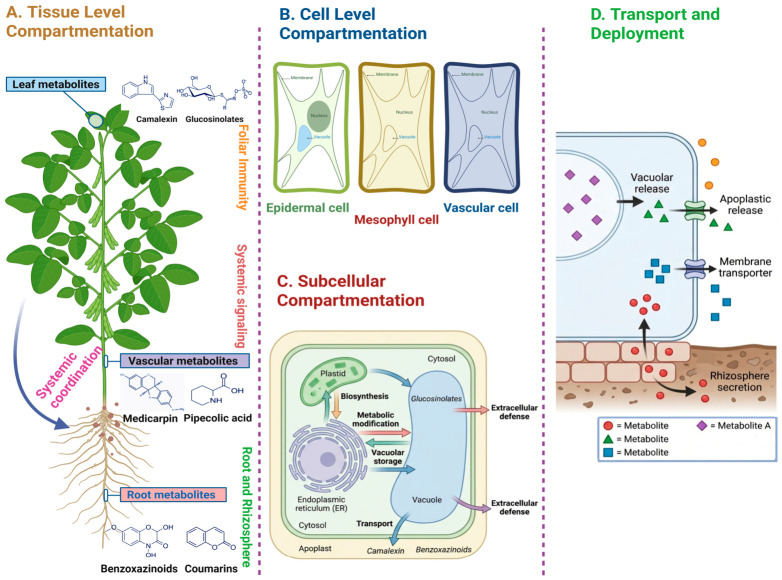
Spatiotemporal compartmentation of specialized metabolites in plant cells, tissues, and subcellular compartments.

Furthermore, extracellular disposition may extend defense metabolism toward ecological space. The specialized metabolites from roots can affect the composition of the microbiome and its functions [73], whereas pathogen or commensal metabolism of such compounds may alter their bioactivity [74]. Consequently, the transport of specialized metabolites should be considered a determinant of specific defense-chemistry expression and relevant biotic (stressor) interactions. Thus, spatiotemporal compartmentation links biosynthesis to biological function by regulating the local concentration, activation timing, and exposure interface [75] (Figure 3).

## 6. From Metabolic Reprogramming to Quantitative Defense Phenotypes

In plant immunity, one of the fundamental challenges is explaining the diverse metabolic reprogramming modes that are essential for converting them into quantifiable resistance phenotypes. This quantitative defense merely reveals the activity of a single metabolite. It arises from the additive and context-dependent effects of multiple compounds and their derivatives, as well as from the regulatory pathways that produce them. The phenotypic interpretation thereof is complicated, as the metabolite accumulation and pathogenic restrictions do not always scale linearly. Therefore, a quantitative framework is mandatory to correlate the resistance, chemistry, and overall plant performance against stressors.

### 6.1. Linking Metabolite Accumulation to Resistance Outcomes

Investigations have presented sufficient evidence to support extensive contributions toward quantitative resistance; however, their effects are characteristically integrated rather than specific [76,77]. In *Arabidopsis*, for example, untargeted metabolomics are linked to a general non-self response toward tryptophan-based specialized metabolism. It was established that this chemically defined response contributes toward protection against *Pseudomonas syringae* [78]. Similarly, the integration of metabolite quantitative trait loci (QTL) in *Brassica napus* resolved clubroot resistance into QTL-specific metabolic modules. Therefore, discrete resistance loci are accompanied by distinct metabolic signatures instead of a single universal defense mechanism [79]. These outcomes support the key understanding that metabolite accumulation is most informative when interpreted in the context of genetics and pathways. Correspondingly, for casual inference, correlation alone is not considered sufficient. It has been observed that fewer metabolic shifts accompany resistance, whereas others amplify susceptibility, compensation, or broader physiological stress [80]. Integrative strategies that link metabolomics to quantitative genetics present a more rigorous approach to identifying resistance-associated metabolites by connecting defense loci with metabolic QTL and phenotypic variation [79,81].

### 6.2. Tradeoffs Between Defense, Growth, and Fitness

The quantitative defense phenotypes are primarily dependent on the resistance gains and costs of the defense mechanisms [82]. As specialized metabolite biosynthesis depends on shared carbon, nitrogen, and energy, enhanced chemical defense generally coincides with reduced growth and altered reproduction [20,83]. This trade-off generally occurs at the metabolic, genetic, and physiological levels. For instance, slightly lowered target of rapamycin (TOR) signaling can enhance resistance to pathogens. However, elevated TOR activity can greatly promote growth at the expense of immunity [84].

Furthermore, empirical investigations have shown that these tradeoffs are not fixed. Disruption of the cellulose synthase-like gene OsCSLD4 in rice can result in marked activation of the defense system, which finally leads to enhanced disease resistance and broad metabolic shifts [85]. However, some circumstances allow partial uncoupling of defense and growth, as seen in *Arabidopsis*, where methyl jasmonate seed priming enhanced pest-tailored defenses, thus indicating that timing, baseline metabolic state, and regulatory fine-tuning can substantially alleviate the costs of formal defense [86]. Quantitative defense phenotypes reflect defensive efficacy and the extent to which plants can absorb or redistribute the associated metabolic costs.

### 6.3. Quantitative Phenotyping and Functional Validation

Robust interpretation of the defense metabolism requires phenotyping approaches that extend beyond symptom scoring [87]. Quantitative defense should ideally be assessed using integrated measurements of pathogen biomass, lesion development, biomass accumulation, reproductive output, and metabolite profiles, thereby allowing the defense benefits and fitness costs to be evaluated together [88,89]. Recent studies combining plant phenotyping with metabolomics have revealed that resistance outcomes can vary considerably across microbial partners and host genotypes, even when gross disease phenotypes appear similar [89]. This reinforces the need for phenotype-rich experimental designs when evaluating metabolic defenses.

Functional validation also increasingly benefits from predictive and system-level approaches. Constraint-based metabolic modeling, such as in potato, showed that the activation of multiple defense pathways incurs measurable growth costs and can reproduce treatment-specific metabolite changes under biotic stress [90]. Such approaches complement genetic and metabolomic analyses by identifying the probable bottlenecks, pathway interactions, and hidden tradeoffs [91]. The quantitative defense phenotypes only become mechanistically informative when metabolite data are linked to genetics, physiology, and formal phenotyping rather than treated as descriptive endpoints.

Importantly, metabolite accumulation should not be equated directly with defense effectiveness, as elevated metabolic levels do not necessarily confer enhanced resistance. Rather, defense-associated metabolism becomes functionally relevant only when deployed in the appropriate spatial, temporal, and physiological context.

## 7. Emerging Tools for Dissecting Defense Metabolism

Recent technological advances in analytical sciences have revolutionized the study of plant defense metabolism, shifting it from descriptive to predictive and mechanistic biology. High-resolution metabolomics, spatially resolved detection platforms, network-based modeling, and integration of dynamic multi-omic tools enable specialized metabolism to be studied from the subcellular to quantitative phenotypic levels [92]. Such tools are important for investigating plant immunity, as the identity of a metabolite alone does not explain the overall defense outcomes. This is primarily reliant on contextual information on the localization, regulation, and connectivity of relevant pathways. Therefore, there is a great need to utilize integrated platforms that can readily resolve causality rather than merely detect associations or linkages.

### 7.1. Metabolomics and Spatial Metabolomics

Mass spectrometry-based metabolomics is a foundational platform in plant defense biology. Such analysis provides a robust assessment of pathogen- and pest-induced metabolic changes in targeted and untargeted workflows. These platforms identify resistance biomarkers and candidate defense pathways. However, the choice of platform strongly affects the metabolite coverage and biological interpretation [93,94]. Although conventional metabolomics affords primary insights into the activation of defense-related pathways, it lacks spatial resolution. This is considered a major limitation in cases where metabolite function depends on tissue or subcellular localization.

Metabolomics becomes most informative when the metabolite profiles are linked to defined immune phenotypes rather than treated as descriptive readouts. For example, untargeted metabolomics in *Arabidopsis* revealed that a general nonself immune response was strongly associated with tryptophan-derived metabolic reprogramming and contributes to *Pseudomonas syringae* resistance [95]. Similarly, the integration of metabolite profiling with quantitative genetics in *Brassica napus* resolved clubroot resistance into QTL-specific metabolic modules. This demonstrates that metabolic signatures can be associated with discrete resistance loci rather than with generic responses to stress [96].

However, bulk metabolomics has important limitations for plant immunity research. Incomplete metabolite annotation, platform-dependent detection bias, and difficulty in distinguishing causal defense metabolites from downstream stress correlates remain major constraints [97]. This limitation is particularly relevant because metabolite function in plant immunity often depends on spatial localization and timing, rather than on abundance alone. Spatial metabolomics addresses part of this gap by enabling tissue-resolved detection of metabolites using imaging-based platforms, and recent studies have highlighted its value for resolving localized metabolic heterogeneity in plant systems [98].

This key limitation can be addressed through spatial metabolomics [99]. Recent studies and methodological reviews published in 2025 further emphasize the growing utility of spatial metabolomics in plant systems, particularly for resolving tissue-level metabolite localization as well as linking chemical heterogeneity with biological function [98,100]. Advanced mass spectral imaging platforms, including matrix-assisted laser desorption ionization and desorption electrospray ionization, enable spatially resolved metabolite detection in plant tissues, allowing direct investigation of “points” of metabolite accumulation rather than inferring their location from bulk extracts [88]. From the perspective of defense metabolism, this is particularly important as localized accumulation frequently regulates whether a compound functions as barrier reinforcement, intracellular signaling, microbial exclusion, or systemic communication [101]. Therefore, spatial metabolomics represents a key methodological step toward linking chemical networks to functional immune architecture.

### 7.2. Multiomics Integration and Network Inference

Single-omics studies remain informative, but they rarely capture the complexity of defense metabolisms. Integration of metabolomics with transcriptomics, proteomics, epigenomics, and phenomics is becoming increasingly necessary to resolve biosynthetic pathways, regulatory hierarchies, and pathway–environment interactions [102]. In specialized metabolism research, multiomics integration is particularly valuable because pathway discovery is often impeded by gene redundancy, enzyme promiscuity, and lineage-specific diversification [103]. Integrative approaches, therefore, improve pathway assignment and biological interpretation.

Recent studies have shown that multiomics integration is most useful when it connects metabolic changes with regulatory structures and defense phenotypes. In maize, combined transcriptomic and metabolomic analyses have revealed the coordinated activation of phenylpropanoid, flavonoid, lignin, and terpenoid pathways together with the repression of photosynthetic functions, thereby indicating that defense metabolism emerges from system-wide reprogramming rather than isolated pathway induction [104]. Similarly, integrative metabolomics–genetics approaches have demonstrated that resistance-associated metabolites can be resolved into distinct metabolic modules linked to specific genomic regions, thereby improving the interpretation of causal resistance mechanisms [96,105].

Furthermore, network-based approaches strengthen this framework by identifying hubs, modules, and regulatory connectivity across immune signaling and metabolism. This is particularly relevant in plant immunity, where defense output reflects the distributed interactions among the perception, hormone crosstalk, transcriptional control, and biosynthetic branches rather than linear cause-and-effect relationships [106]. However, multiomics integration remains constrained by differences in temporal resolution, sampling scale, and data harmonization across platforms. Moreover, correlation across omics layers does not in itself establish causality, and many inferred associations remain hypothesis-generating unless supported by genetics, flux analysis, or functional validation [107].

### 7.3. Priorities for Causal and Predictive Models

A key priority is to advance beyond descriptive omics data toward approaches that enable mechanistic understanding and predictive modeling, as the current datasets primarily capture correlations without resolving associations with resistance, fitness tradeoffs, and spatiotemporal variation [108]. Predictive models will therefore require standardized workflows, improved metabolite annotation, isotope-based flux analysis, and experimental validation across the genetic and environmental contexts [109]. Without such advances, defense metabolism will remain easier to profile than to comprehensively explain.

A promising direction is the integration of metabolomics with phenotyping, genetics, and computational modeling [110]. Recent plant system studies have reported that large-scale resources, network frameworks, and compartment-aware models can substantially improve the interpretation of complex metabolic phenotypes [92,111]. In addition, the most beneficial future models for plant immunity will likely be those that predict when and where specific metabolic modules contribute to resistance, how they interact with growth and environment, and which nodes are causal rather than correlative. This shift from descriptive omics to predictive defense metabolism will likely define the next phase of the field (Table 2).

## 8. Conclusions and Future Directions

Specialized metabolic reprogramming is the foundation of plant immunity by linking pathogen perception to the synthesis and spatial deployment of chemical defenses and the regulation of quantitative resistance. The evidence reviewed herein indicates that defense metabolism operates as an integrated, dynamically regulated network that is shaped by biosynthetic branching, hormone crosstalk, cellular and subcellular compartmentation, and resource allocation. Within this framework, immune perception activates signaling modules that prioritize the transcriptional and hormonal responses, redirect metabolic flux, and control the spatial deployment of defense metabolites. These coordinated processes determine the defense phenotype by shaping resistance outcomes, together with associated growth and fitness tradeoffs. Therefore, defense effectiveness depends not only on metabolite composition but also on metabolite accumulation timing, location, and metabolic context. A priority for future research is to extend beyond descriptive metabolite profiling toward causal and predictive frameworks. This will require the tighter integration of metabolomics, spatial metabolomics, genetics, phenotyping, and computational modeling, along with improved metabolite annotation, flux-resolved analyses, and compartment-aware experimental designs. Identifying causal metabolic nodes, defining how defense chemistry is prioritized under growth-limited conditions, and elucidating how localized metabolite pools contribute to whole-plant immune performance will enhance our mechanistic understanding and crop development with durable, resource-efficient immunity.

## Figures and Tables

**Table 1:** Major classes of specialized metabolites involved in plant immunity, with representative biosynthetic origins and immune-associated functions.

Metabolite Class/Representative Systems	Representative Compounds	Pathway Origin	Key Genes/Enzymes	Main Immune Roles	Biotic Context	Ref
Indolic phytoalexins (*Arabidopsis*)	Camalexin and ICA derivatives	Tryptophan	CYP71A12, CYP71A13, and PAD3	Antimicrobial defense	Foliar pathogens	[18,19]
Glucosinolates (*Arabidopsis, Brassica*)	Indolic and aliphatic glucosinolates	Amino acid-derived	CYP79, CYP83, GSTs, and myrosinases	Defense, signaling, and tradeoffs	Pathogens, herbivores	[20,33]
Benzoxazinoids (maize, wheat, and rye)	DIBOA, DIMBOA, and MBOA	Indole-derived	BX1-BX8	Antimicrobial and rhizosphere defense	Root pathogens and herbivores	[22,23]
Phenylpropanoids (broadly distributed)	Lignin precursors, flavonoids, and coumarins	Phenylalanine	PAL, C4H, 4CL, and CHS	Cell wall defense and redox buffering	Fungi and wound stress	[1,7]
Terpenoids (broadly distributed)	Mono, sesqui, and diterpenoids	Isoprenoid pathways	TPSs, P450S	Direct and indirect defense	Herbivores, pathogens	[37,38]
Lysine-derived metabolites of *Arabidopsis*	Pipecolic acid and NHP	Lysine catabolism	ALD1, SARD4, and FMO1.	SAR, priming	Systemic infection	[24,25]
Alkaloids (lineage-specific taxa)	Diverse alkaloids	Variable	Taxon-specific	Chemical defense	Herbivores, pathogens	[1,39]

**Table 2:** Emerging analytical platforms for investigating defense metabolism in plants.

Approach/Main Use	Primary Output	Main Strength	Main Limitation	Best Use in Plant Immunity	Ref
Untargeted metabolomics/broad profiling	Global metabolomic patterns	Wide coverage	Limited annotation	Pathway discovery and biomarker screening	[112]
Targeted metabolomics/precision quantification	Defined metabolite levels	High sensitivity	Narrow scope	Validation of defense metabolites	[113]
Spatial metabolomics/tissue localization	Spatial metabolite maps	Positional resolution	Technical complexity	Tissue-specific defense chemistry	[114]
Transcriptomics + metabolomics/coregulation	Gene–metabolite modules	Pathway inference	Correlation only	Regulatory pathway mapping	[115]
Multiomics integration/system analysis	Cross-layer networks	Mechanistic depth	High data complexity	Integrative defense analysis	[116]
Network inference/module detection	Hubs, modules, and edges	System organization	Model dependence	Immune network structure	[117]
Genetic metabolomics/QTL linkage	Metabolic QTLs	Causal linkage	Population dependent	Resistance–metabolite associations	[118]
Constraint-based modeling/predictive analysis	Flux and trade-off predictions	Hypothesis generation	Requires robust models	Growth–defense tradeoffs	[119]

## Data Availability

Data are contained within the article.

## Abbreviations

4CL, 4-Coumarate:CoA ligase; ABA, Abscisic acid; ALD1, AGD2-Like Defense Response Protein 1; AP2, APETALA2; bHLH, Basic helix–loop–helix; bZIP, Basic leucine zipper; C4H, Cinnamate 4-hydroxylase; CHS, Chalcone synthase; CYP, Cytochrome P450; CYP71A12, Cytochrome P450 71A12; CYP71A13, Cytochrome P450 71A13; CYP79, Cytochrome P450 79; CYP83, Cytochrome P450 83; DIBOA, 2,4-Dihydroxy-1,4-benzoxazin-3-one; DIMBOA, 2,4-Dihydroxy-7-methoxy-1,4-benzoxazin-3-one; DNA, Deoxyribonucleic acid; ER, Endoplasmic reticulum; ERF, Ethylene response factor; ET, Ethylene; FMO1, Flavin-dependent monooxygenase 1; GSTs, Glutathione S-transferases; HRM, High-resolution metabolomics; JA, Jasmonic acid; JAZ, Jasmonate ZIM-domain; MALDI, Matrix-assisted laser desorption/ionization; MBOA, 6-Methoxy-benzoxazolin-2-one; MYB, MYB transcription factor; MYC, MYC transcription factor; NAC, NAM, ATAF1/2, and CUC2; NHP, N-Hydroxypipecolic acid; ORA59, Octadecanoid-Responsive Arabidopsis AP2/ERF 59; PAD3, Phytoalexin-Deficient 3; PAL, Phenylalanine ammonia-lyase; QTL, Quantitative trait locus; RNA, Ribonucleic acid; ROS, Reactive oxygen species; SA, Salicylic acid; SAR, Systemic acquired resistance; SARD4, Systemic Acquired Resistance Deficient 4; TCA, Tricarboxylic acid; TOR, Target of rapamycin; WRKY, WRKY transcription factor.
